# Genetic and Functional Evidence of Complement Dysregulation in Multiple Myeloma Patients with Carfilzomib-Induced Thrombotic Microangiopathy Compared to Controls

**DOI:** 10.3390/jcm11123355

**Published:** 2022-06-10

**Authors:** Eleni Gavriilaki, Dimitra Dalampira, Foteini Theodorakakou, Christine-Ivy Liacos, Nikolaos Kanellias, Evangelos Eleutherakis-Papaiakovou, Evangelos Terpos, Maria Gavriatopoulou, Evgenia Verrou, Theodora Triantafyllou, Aggeliki Sevastoudi, Evaggelia-Evdoxia Koravou, Tasoula Touloumenidou, Christos Varelas, Apostolia Papalexandri, Ioanna Sakellari, Meletios A. Dimopoulos, Efstathios Kastritis, Eirini Katodritou

**Affiliations:** 1 Hematology Department—BMT Unit, G. Papanicolaou Hospital, 570 10 Thessaloniki, Greece; elenicelli@yahoo.gr (E.G.); evakikor@gmail.com (E.-E.K.); tasoula.touloumenidou@gmail.com (T.T.); varelaschris@gmail.com (C.V.); lila.papalexandri@gmail.com (A.P.); bmt@gpapanikolaou.gr (I.S.); 2 Hematology Department, Theagenio Cancer Hospital, 546 39 Thessaloniki, Greece; everrou@gmail.com (E.V.); dora_triant@hotmail.com (T.T.); medsev667@gmail.com (A.S.); eirinikatodritou@gmail.com (E.K.); 3 Department of Clinical Therapeutics, National and Kapodistrian University of Athens, 115 28 Athens, Greece; foteint@gmail.com (F.T.); liakou@med.uoa.gr (C.-I.L.); nick.kanellias@gmail.com (N.K.); mdeleutherakis2@gmail.com (E.E.-P.); eterpos@med.uoa.gr (E.T.); mariagabria@gmail.com (M.G.); mdimop@med.uoa.gr (M.A.D.); ekastritis@gmail.com (E.K.)

**Keywords:** carfilzomib, complement, thrombotic microangiopathy

## Abstract

Background: Carfilzomib, an irreversible proteasome inhibitor approved for the treatment of relapsed/refractory Multiple Myeloma (MM) has been associated with Thrombotic Microangiopathy (TMA). Several pathogenetic mechanisms of carfilzomib-induced TMA have been proposed; however, recently, there has been a shift of focus on the potential contribution of complement dysregulation. Our aim was to explore whether patients with carfilzomib-induced TMA harbor germline variants of complement-related genes, which have been characterized as risk factors for TMA. Methods: We retrospectively recruited consecutive MM patients with carfilzomib-induced TMA and compared them to MM patients who received ≥4 cycles of carfilzomib and did not develop signs/symptoms of TMA, in a 1:2 ratio. Genomic DNA from peripheral blood was analyzed using next generation sequencing (NGS) with a complement-related gene panel; ADAMTS13 activity and soluble C5b-9 were measured using ELISA. Results: Complement-related variants were more common in patients with carfilzomib-induced TMA compared to non-TMA controls, regardless of patient and treatment characteristics; ADAMTS13 activity and C5b-9 were compatible with the phenotype of complement-related TMA. Conclusions: We confirmed the previous findings that implicated complement-related genes in the pathogenesis of carfilzomib-induced TMA. Most importantly, by incorporating a control group of non-TMA MM patients treated with carfilzomib-based regimens and functional complement assays, we enhanced the credibility of our findings.

## 1. Introduction

Multiple Myeloma (MM) is a plasma cell dyscrasia, accounting for ~10% of all hematologic neoplasms, which is characterized by extreme proliferation of plasma cells secreting monoclonal protein [1]. Among other drugs, proteasome inhibitors (PIs) including bortezomib, carfilzomib and ixazomib have been incorporated into the treatment of both newly diagnosed and relapsed/refractory MM (RRMM) during the last years [2]. Carfilzomib is a tetrapeptide epoxyketone second-generation irreversible PI, administered intravenously, that selectively binds the β5 subunit exhibiting chymotrypsin-like activity, and in higher concentrations, displays trypsin-like activity. The drug was approved in the United States of America in 2012 and in Europe in 2015 for patients with RRMM [3,4]. The initial trials that led to FDA approval of bortezomib and carfilzomib did not report thrombotic microangiopathy (TMA) as a drug-related adverse event. However, several case reports and series have identified TMA as an adverse effect of PIs, with more reports relating this complication to carfilzomib rather than to the other PIs.

Thrombotic microangiopathies represent a heterogeneous group of syndromes that are characterized by the clinical including microangiopathic hemolytic anemia, thrombocytopenia, and organ damage [5]. Two major entities with distinct pathophysiology have been recognized: thrombotic thrombocytopenic purpura (TTP) caused by ADAMTS13 (a disintegrin and metalloproteinase with a thrombospondin type 1 motif, member 13) and atypical hemolytic uremic syndrome (aHUS) caused by complement dysregulation. Furthermore, TMAs associated with underlying conditions, such as drugs, malignancy, infections [6], have been also recognized. Understanding the discrete pathophysiological mechanisms involved in TMAs is crucial in order to manage patients successfully.

The proposed pathogenesis of PIs-induced TMA includes immune-mediated and dose-dependent toxicity mechanisms [7]. A link between PIs’ action and Vascular Endothelial Growth Factor (VEGF) inhibition via NF-kB pathways causing microvascular injury to the glomerular capillaries has been reported. Moreover, it has been suggested that for potential immune-mediated mechanisms for PI-associated TMA, PIs may induce high levels of proinflammatory cytokines such as IL-6 and TNF-a, which may facilitate the formation of autoantibodies directed at ADAMTS13. Recently, it has been recognized that a complement may play a central role in the pathogenesis of several subcategories of TMA, including drug-induced TMA (DITMA) [7]. A limited number of studies has addressed the importance of a complement in DITMA using clinical evidence, functional or genetic assays, including next-generation sequencing (NGS). However, the exact contribution of a complement in DITMA, and particularly carfilzomib-induced TMA, has not yet been elucidated.

Given the limited data in the field, we sought to evaluate the possible presence of complement-related variants in patients with carfilzomib-induced TMA and their potential role, compared to a control group of non-TMA patients treated with carfilzomib-based regimens.

## 2. Experimental Section

### 2.1. Patient Population

We retrospectively recruited consecutive MM patients that had developed carfilzomib-induced TMA. As a control group, we studied MM patients that had received at least 4 cycles of carfilzomib treatment and had no history of signs/symptoms of TMA, in a 1:2 ratio. All patients were of Caucasian origin. TMA was diagnosed based on classical TMA criteria (new-onset or progressive thrombocytopenia, microangiopathic hemolytic anemia and organ damage including acute kidney injury and/or microvascular thrombosis on tissue biopsy), in the absence of disease progression. The study was approved by our Institutional Review Board and was conducted in accordance with the Helsinki Declaration. All patients provided written informed consent to participate in the study.

### 2.2. Genetic Analysis 

Genomic DNA was isolated from peripheral blood samples and was analyzed using next generation sequencing (NGS) with a customized complement-related gene panel (complement factor H/*CFH, CFH-related, CFI, CFB, CFD, C3, CD55, C5, CD46, thrombomodulin/THBD*), including TMA-associated *ADAMTS13 (A Disintegrin and Metalloproteinase with Thrombospondin motifs)*. Probes were designed using Design Studio (Illumina, San Diego, CA, USA) to cover all exons spanning 15 bases into the intronic regions (98% coverage). We used 10 ng of initial DNA material. Libraries were quantified using Qubit and were sequenced on a MiniSeq System in a 2 × 150 bp run (Illumina, San Diego, CA, USA). Reported variant allele frequency was set at ≥20%. High quality of the sequencing was confirmed using fastqc (v0.11.5) [8]. Both Ensembl and Refseq resources were used for annotation of the output files. Variants’ clinical significance was based on ClinVar. Variants were reported based on standards and guidelines for the interpretation of variants with the use of specific standard terminology—“pathogenic”, “likely pathogenic”, “uncertain significance”, “likely benign” and “benign” [9]. In particular, complement-related variants have been evaluated based on previous relevant validations [10].

### 2.3. Functional Analysis

EDTA plasma and sera were isolated from patients at TMA diagnosis and were stored immediately at −80 °C. Aliquots were thawed only once to avoid multiple freeze–thaw cycles. ADAMTS13 activity and soluble C5b-9 were measured using commercially available ELISA kits (Technozym Diapharma, Louisville, KY and Quidel, San Diego, CA, USA) in sera and EDTA plasma, respectively, since these were the only available samples from this rare set of patients Although ADAMTS13 measurement is recommended on sodium citrate plasma samples, our laboratory compared ADAMTS13 activity in sera and plasma samples (sodium citrate) of other patient populations and found similar results [11].

### 2.4. Statistical Analysis

Data were analyzed using the statistical program SPSS 22.0 (IBM SPSS Statistics for Windows, Version 22.0. IBM Corp, Armonk, NY, USA). Continuous variables were described as median and range and categorical variables as frequencies. Variables were compared using chi-square and one-way ANOVA for categorical variables and Mann–Whitney for continuous variables.

## 3. Results

### 3.1. Patient Population

The study enrolled 13 ΜΜ patients with a history of carfilzomib-induced TMA and 26 control MM patients. The characteristics of patients that developed TMA and of control myeloma patients at the start of carfilzomib treatment are summarized in Table 1. There were no significant differences in the baseline characteristics between the two groups, except from platelet count, which was marginally higher in controls (*p* < 0.05); bone marrow infiltration was marginally higher in the TMA group, although this difference did not reach statistical significance (*p* = 0.15).

At the time of diagnosis of TMA, all patients presented with evidence of microangiopathic hemolytic anemia (detection of schistocytes in the peripheral blood, increased LDH, substantial reduction of serum haptoglobin, and decreased platelet counts) and all developed severe renal dysfunction (median eGFR at the diagnosis of TMA was 20.5 mL/min/1.73 m^2^, range 3–53 mL/min/1.73 m^2^). In four patients, the diagnosis was confirmed by renal biopsy (all four had also newly developed non-specific proteinuria). At the time of development of TMA, all patients were in myeloma remission (Table 1).

Neither the median number of previous lines nor the median dose of carfilzomib did not differ between groups (*p* > 0.05). Carfilzomib was administered twice weekly or once weekly in 11/13 vs. 14/26 and 2/13 vs. 12/26 of TMA patients vs. non TMA, respectively (*p* < 0.05). The median duration of carflizomib administration was longer in controls compared to TMA patients (Table 1).

Carfilzomib was discontinued in all 13 patients that developed TMA; four patients were treated with supportive care including hydration, steroids and fresh frozen plasma infusion, and in nine patients, plasma exchange was used with or without steroids. In one patient with refractory TMA, rituximab was added to the regimen, based on published cases series [12]. One patient developed multiorgan failure as a result of superimposed bacterial sepsis, while the patient who had TMA refractory to plasma exchange and steroids and received rituximab was subsequently complicated by CMV infection. In 11/13 patients, renal function and platelet counts were restored. One patient restarted carfilzomib after the resolution of TMA without experiencing TMA recurrence. None of our patients were treated with eculizumab, as TMA resolved with conventional management; 2/13 patients maintained moderately abnormal eGFR (40 and 36 mL/min, respectively) without progressing to end stage renal disease.

### 3.2. Genetic Analysis

A total of 381 variants were identified, while the number of variants per patient ranged from 46 to 91 with a mean value of 68 and standard deviation of 9. Given that most complement-related variants do not have a clinical annotation, we further discuss only variants that are annotated as risk factors possibly pathogenic. Details on the described variants are shown in Table 2.

We found a variant of *ADAMTS13* (rs2301612; missense; previously described in congenital TTP [17]) in 8 out of 13 (61.5%) TMA patients; compared to 9/26 (34.6%) controls (*p* = 0.11). We also detected two missense risk factor variants, previously described in complement-related diseases: rs2230199 in *C3* (5/13 (38.4%) TMA patients vs. 5/26 (19.2%) controls; *p* = 0.19)) [18]; and rs800292 in *CFH* (6/13 (46.1%) TMA patients; vs. 9/26 (34.6%) controls *p* = 0.39)) [19]. Among them, 6/13 (46.1%) TMA patients had a combination of these characterized variants, which was significantly more common in TMA patients compared to controls (6/13 (46%) vs. 4/26 (15.3%), *p* = 0.019). At least one complement-related variant was found in all TMA patients compared to 18/26 (69.2%) controls (*p* = 0.02). Furthermore, one patient with TTP had a rare germline missense variant in *CFI* (rs112534524), previously detected in Ahus [14]. The results are summarized in Table 3

### 3.3. Functional Analysis

To further determine the interaction between genotype and phenotype, we also measured ADAMTS13 and soluble C5b-9 in four patients with available samples. Median ADAMTS13 values were 67.5% (range 48–95), with a normal range set at 80–120% by the manufacturer. Median C5b-9 values were increased in all patients with carfilzomib-induced TMA (309 ng/mL, range 289–320, with an upper normal limit set at 245 ng/mL for measurements in our laboratory). These findings are compatible with the phenotype of complement-related TMA.

## 4. Discussion

Our study provides genetic and functional evidence of complement overactivation in the largest series of carfilzomib-induced TMA as of today. We also show for the first time that complement-related variants are more common in patients with carfilzomib-induced TMA compared to non-TMA controls that have received similar doses of carfilzomib.

Drug-induced TMA is a well-recognized clinical entity in patients with MM treated with PIs and more profoundly with carfilzomib [20]. Several case reports and series have already described the phenomenon [21,22,23,24]. Yui et al. have reported the largest series thus far, including eleven patients diagnosed with PI-induced TMA, eight of which were treated with carfilzomib. In this study, other causes of TMA including inhibition of ADAMTS13, malignancy and other medication were ruled out; importantly, nine patients had resolution of TMA after withdrawal of PI, and one patient had recurrence of TMA with rechallenge of PI.

The exact mechanisms contributing to the development of PI-induced TMA are not yet clear. Both immune-mediated and dose-dependent toxicity mechanisms have been identified [20]. This setting is complicated by co-administration of partner drugs (e.g., daratumumab or lenalidomide) or dexamethasone as part of MM treatment that may influence the severity of the manifestations and TMA outcome. Previous reports have shown that immediate discontinuation of carfilzomib plus supportive care may be sufficient alone to improve the disease’s manifestations [24,25,26]. Furthermore, co-administration of other drugs, and most importantly dexamethasone, has reduced the incidence of carfilzomib-induced TMA or severity of its clinical manifestations [27]. It has been demonstrated that eculizumab, the first-in-class monoclonal antibody against the complement protein C5, could be beneficial in patients with PIs-induced TMA [28,29]. Eculizumab has shown long-term safety and efficacy in patients with complement-mediated disease, including complement-mediated TMAs [30]. Therefore, the reports of efficacy in carfilzomib-induced TMA suggests that dysregulation of complement activation may have a central role in the mechanisms of this complication [24].

Despite clinical evidence, mechanistic evidence is limited. Blasco et al. have recently evaluated the potential role of terminal complement pathway in four MM patients diagnosed with carfilzomib-induced TMA, by measuring the membrane attack complex (C5b-9) deposition on endothelial cells in culture exposed to plasma from patients during the acute phase of TMA. The results revealed complement overactivation as a mechanism of potential endothelial damage in three out of four patients [31]. Although this assay is useful for mechanistic studies, its usefulness and accessibility in clinical diagnostics are rather limited. Therefore, we studied soluble C5b-9 levels that are more easily accessible and have been proven as useful clinical biomarkers in other complement-mediated TMAs [32]. Our findings of elevated C5b-9 in four out of four patients studied strongly support complement dysregulation in the plasma of patients with carfilzomib-induced TMA.

Genetic assays and most importantly NGS have also been used to evaluate the implication of a complement in the pathogenesis of carfilzomib-related TMA [33]. In a case report, Portuguese and Lipe reported three patients with carfilzomib-induced aHUS. Both genetic and functional complement pathway assays were performed, and two out of three patients were found to harbor heterozygous CFHR3-CFHR1 deletions, while CFH autoantibodies were negative. Functional alternative complement pathway testing was unremarkable in all three patients but cannot be evaluated since it was performed after remission of acute TMA [34]. It has been postulated that homozygous deletion encompassing CFH-related protein genes observed in 5.7% of European–American and Finnish descent were associated with aHUS, whereas heterozygous deletion is generally considered as a common benign variant [35]. However, in the aforementioned study, Portuguese and Lipe supported that the administration of carfilzomib may “switch on” a benign variant (i.e., heterozygous CFHR3-CFHR1 deletion) to a conditionally functional mutation leading to the development of aHUS [34]. This hypothesis is reasonable, based not only on the “two-hit” hypothesis of aHUS, but also on findings of complement-related variants identified as risk factors in other disorders with complement involvement, such as **Coronavirus Disease 2019 (COVID-19)** [36,37]. In a recently reported 10-patient cohort, Moscvin et al. examined the genetic variants in patients diagnosed with carfilzomib-induced TMA: deletions of the CFHR3-CFHR5 region were present in seven cases (70%) [38]. Our report is also the first one to investigate ADAMTS13 variants in this patient population. ADAMTS13 variants have been described in patients with complement-mediated TMAs [11,39]. Similar to our study, these patients also have partially reduced ADAMTS13 activity. Therefore, we believe that these data need to be documented in these first reports of the genetic background of these patients.

Despite the interesting observations that correlate carfilzomib-induced TMA with certain variants of complement-related genes, none of the previously mentioned studies explored the possible differences in the mutational status of complement-related genes between patients exhibiting TMA and a control group of MM patients treated with carfilzomib-based regimens without developing TMA. In the current study, we found complement-related variants in all our patients who developed TMA and when comparing to the control group consisting of MM patients treated similarly, but never developing TMA, we found significant differences. The selection of this control group provides an important advantage of eliminating potential differences due to the underlying disease (MM) and treatment. This is of great value since most TMA patients harbored complement-related variants that are considered risk factors for complement-related diseases but are not categorized as rare. Our population also has the advantage of homogeneous origin (Caucasian), which is important to extract safe results for complement-related variants. The homogeneity of our population is evident by the fact that complement-related variants in the control group were found in similar frequencies to those reported for Caucasian populations [15,16,17,18].

Finally, our study investigated the genetic and functional fingerprint of complement dysregulation in carfilzomib-induced TMA, aiming to provide a background for the potential use of a complement inhibitor in patients that do not respond to first-line treatment. The majority of our patients responded to first-line treatment and had restored renal function. Although eculizumab was not used in our patients, data from the prototype disease (aHUS) suggest that renal dysfunction might be reversible with complement inhibition. In this context, several complementopathies share common characteristics with aHUS [5]. Nevertheless, the phenotype and course of the disease are not identical. For example, functional and genetic evidence of complement dysregulation have also been shown in patients with catastrophic APS [40]. In the case of drug-induced TMA, the drug might be the trigger of this situation. Similar to other triggers (inflammation, surgery, etc.), re-exposure to the trigger does not necessarily lead to TMA manifestation. Although in our study the drug was restarted only in one patient, without recurrence of TMA, further data are necessary to draw safe conclusions.

Our study has some limitations. First, we were not able to combine functional and genetic data in all patients. Second, our results are limited by known hurdles in interpretation of NGS findings of complement-related diseases that have also been previously recognized by our group [11]. Accumulation of genetic data in complement-related disease considering both common and rare variants is needed to overcome these limitations. Furthermore, our NGS panel includes CFH-related genes but does not have high specificity in detecting deletions or insertions. Given that factor-H autoantibodies have not been detected in previous studies of carfilzomib-induced TMA [34,38], the pathogenetic role of these findings needs to be carefully assessed. In addition, our data cannot directly link carfilzomib-induced TMA to complement-mediated TMAs. Our patients present with an acute, non-relapsing form of TMA, with favorable kidney outcomes and genetic variants recognized as TMA risk factors, contrasting complement-mediated TMA characterized by severe kidney disease, high relapse rate, poor kidney outcomes if left untreated, and high prevalence of rare and pathogenetic variants. Lastly, our data need to be verified by additional studies to provide implications for the use of genetic testing for early identification of patients with TMA. Therefore, the usefulness of pre-treatment screening to assess the risk of TMA has not been elucidated yet.

## 5. Conclusions

In conclusion, carfilzomib-induced TMA shares common genetic and phenotypic characteristics with complement-related TMAs. Given the role of treatment with carfilzomib in myeloma, increased awareness is needed by treating physicians for carfilzomib-induced TMA to early diagnose and manage this potentially life-threatening syndrome. Further studies are also warranted to delineate the role of complement inhibitors in selected patient populations that are expected to benefit from specific treatment.

## Figures and Tables

**Table 1:** Patient and treatment characteristics.

Variable	TMA Patients (*n* = 13)	Non-TMA Patients (*n* = 26)	*p*
**Patient characteristics**			
Age	62 (44–69)	62 (41–76)	NS
Sex	M: 6, F: 7	M: 8, F: 18	NS
M-Component	IgG: 10, IgA: 2, LC: 1	IgG: 17, IgA: 5, LC:4	NS
Hb (g/dL)	11.6 (7.6–14.8)	12.2 (8.1–15.9)	NS
PLT (×10^3^/μL)	163 (49–282)	243 (76–447)	0.04
EGFR (CKD-EPI)	77.9 (35.6–123.7)	80 (50–117)	NS
ISS at carfilzomib administration	ISS1: 5, ISS2: 6, ISS3: 2	ISS1: 12, ISS2: 12, ISS3: 2	NS
β2 Microblobulin (mg/L)	3.5 (2.00–6.35)	4.00 (1.4–0.00)	NS
LDH (U/L) **Bone marrow infiltration % (range)**	**132 (125–276)** **60 (51–90)**	**148 (96–231)** **39 (8–88)**	NS NS
**Treatment characteristics**			
Line of therapy	1st: 1, 2nd: 8, 3d: 3. 4th: 1	1st: 12, 2nd: 6, 3d: 3, 4th: 2 beyond 4th: 3	NS
Carfilzomib-based regimen	Dara-Kd: 4, Kd: 6, KRd: 3	Dara-Kd 8, Kd: 1, KRd: 12, KCd: 2, KPd: 3	NS
Median Carfilzomib dose per infusion (mg)	100 (56–115)	102 (27–124)	NS
Median duration of carfilzomib administration (months)	5 (0.5–63)	12 (5–57)	<0.001
Response	≥vgPR: 7, PR: 5, PD: 1	≥vgPR: 22, PR:4	0.02

Hb: hemoglobin; PLT: platelets; eGFR: estimated glomerular filtration rate; ISS: International staging system; LDH: lactate dehydrogenase; NS: non-significant.

**Table 2:** Genetic variants detected in TMA patients that have previously been reported as pathogenic or risk factors in other TMAs according to the ClinVar database.

Chr	Gene	Reference SNV	CLINSIG	Effect	Syndrome	Reference
Chr9	ADAMTS13	rs2301612	Pathogenic	Missense	Congenital TTP	[13]
Chr4	CFI	rs112534524	Pathogenic	Missense	aHUS	[14]
Chr19	C3	rs2230199	Risk factor	Missense	aHUS, AMD	[15] (total 149)
Chr1	CFH	rs800292	Risk factor	Missense	aHUS, AMD, MPGN	[16] (total 298)

Chr: chromosome; CLINSIG: clinical significance in ClinVar; TTP: thrombotic thrombocytopenic purpura; Ref: references; aHUS: atypical hemolytic uremic syndrome; MPGN: membranoproliferative glomerulonephritis; AMD: age-related macular degeneration; SNV: single nucleotide variation.

**Table 3:** Complement-related genes in TMA patients vs. controls.

Gene	TMA Patients	Controls	*p*
ADAMTS13	8/13 (61.5%)	9/26 (34.6%)	0.11
C3	5/13 (38.4%)	5/26 (19.2%)	0.19
CFH	6/13 (46.1%)	9/26 (34.6%)	0.39
≥1 variant	13/13 (100%)	18/26 (69.2%)	0.02
≥2 variants	6/13 (46.1%)	4/26 (15.4%)	0.03

## Data Availability

Data are available upon request to corresponding author.

## References

[1] Rajkumar S.V. (2020). Multiple myeloma: 2020 update on diagnosis, risk-stratification and management. Am. J. Hematol..

[2] Ito S. (2020). Proteasome Inhibitors for the Treatment of Multiple Myeloma. Cancers.

[3] Vij R., Siegel D.S., Jagannath S., Jakubowiak A.J., Stewart A.K., McDonagh K., Bahlis N., Belch A., Kunkel L.A., Wear S. (2012). An open-label, single-arm, phase 2 study of single-agent carfilzomib in patients with relapsed and/or refractory multiple myeloma who have been previously treated with bortezomib. Br. J. Haematol..

[4] Siegel D.S., Martin T., Wang M., Vij R., Jakubowiak A.J., Lonial S., Trudel S., Kukreti V., Bahlis N., Alsina M. (2012). A phase 2 study of single-agent carfilzomib (PX-171-003-A1) in patients with relapsed and refractory multiple myeloma. Blood.

[5] Gavriilaki E., Brodsky R.A. (2020). Complementopathies and precision medicine. J. Clin. Investig..

[6] Chatzikonstantinou T., Gavriilaki M., Anagnostopoulos A., Gavriilaki E. (2020). An update in drug-induced thrombotic microangiopathy. Front. Med..

[7] Portuguese A.J., Gleber C., Passero F.C., Lipe B. (2019). A review of thrombotic microangiopathies in multiple myeloma. Leuk. Res..

[8] Andrews S. Fast QC: A Quality Control Tool for High throughput Sequence Data. https://www.bioinformatics.babraham.ac.uk/projects/fastqc/.

[9] Richards S., Aziz N., Bale S., Bick D., Das S., Gastier-Foster J., Grody W.W., Hegde M., Lyon E., Spector E. (2015). Standards and guidelines for the interpretation of sequence variants: A joint consensus recommendation of the American College of Medical Genetics and Genomics and the Association for Molecular Pathology, Genetics in medicine. Off. J. Am. Coll. Med. Genet..

[10] Osborne A.J., Breno M., Borsa N.G., Bu F., Frémeaux-Bacchi V., Gale D.P., van den Heuvel L.P., Kavanagh D., Noris M., Pinto S. (2007). Statistical validation of rare complement variants provides insights into the molecular basis of atypical hemolytic uremic syndrome and C3 glomerulopathy. J. Immunol..

[11] Gavriilaki E., Touloumenidou T., Sakellari I., Batsis I., Mallouri D., Psomopoulos F., Tsagiopoulou M., Koutra M., Yannaki E., Papalexandri A. (2020). Pretransplant genetic susceptibility: Clinical relevance in transplant-associated thrombotic microangiopathy. Thromb. Haemost..

[12] Epperla N., Hemauer K., Hamadani M., Friedman K.D., Kreuziger L.B. (2017). Impact of treatment and outcomes for patients with posttransplant drug-associated thrombotic microangiopathy. Transfusion.

[13] Arning A., Jeibmann A., Köhnemann S., Brokinkel B., Ewelt C., Berger K., Wellmann J., Nowak-Göttl U., Stummer W., Stoll M. (2016). ADAMTS genes and the risk of cerebral aneurysm. J. Neurosurg..

[14] Nilsson S.C., Karpman D., Vaziri-Sani F., Kristoffersson A.C., Salomon R., Provot F., Fremeaux-Bacchi V., Trouw L.A., Blom A.M. (2007). A mutation in factor I that is associated with atypical hemolytic uremic syndrome does not affect the function of factor I in complement regulation. Mol. Immunol..

[15] Valoti E., Alberti M., Iatropoulos P., Piras R., Mele C., Breno M., Cremaschi A., Bresin E., Donadelli R., Alizzi S. (2019). Rare Functional Variants in Complement Genes and Anti-FH Autoantibodies-Associated aHUS. Front. Immunol..

[16] Cascella R., Strafella C., Longo G., Manzo L., Ragazzo M., De Felici C., Gambardella S., Marsella L.T., Novelli G., Borgiani P. (2018). Assessing individual risk for AMD with genetic counseling, family history, and genetic testing. Eye.

[17] Kokame K., Matsumoto M., Soejima K., Yagi H., Ishizashi H., Funato M., Tamai H., Konno M., Kamide K., Kawano Y. (2002). Mutations and common polymorphisms in ADAMTS13 gene responsible for von Willebrand factor-cleaving protease activity. Proc. Natl. Acad. Sci. USA.

[18] Zhang J., Li S., Hu S., Yu J., Xiang Y. (2018). Association between genetic variation of complement C3 and the susceptibility to advanced age-related macular degeneration: A meta-analysis. BMC Ophthalmol..

[19] Hageman G.S., Anderson D.H., Johnson L.V., Hancox L.S., Taiber A.J., Hardisty L.I., Hageman J.L., Stockman H.A., Borchardt J.D., Gehrs K.M. (2005). A common haplotype in the complement regulatory gene factor H (HF1/CFH) predisposes individuals to age-related macular degeneration. Proc. Natl. Acad. Sci. USA.

[20] Yui J.C., Van Keer J., Weiss B.M., Waxman A.J., Palmer M.B., D’Agati V.D., Kastritis E., Dimopoulos M.A., Vij R., Bansal D. (2016). Proteasome inhibitor associated thrombotic microangiopathy. Am. J. Hematol..

[21] Qaqish I., Schlam I.M., Chakkera H.A., Fonseca R., Adamski J. (2016). Carfilzomib: A cause of drug associated thrombotic microangiopathy, Transfusion and apheresis science. Off. J. World Apher. Assoc. Off. J. Eur. Soc. Haemapher..

[22] Sullivan M.R., Danilov A.V., Lansigan F., Dunbar N.M. (2015). Carfilzomib associated thrombotic microangiopathy initially treated with therapeutic plasma exchange. J. Clin. Apher..

[23] Lodhi A., Kumar A., Saqlain M.U., Suneja M. (2015). Thrombotic microangiopathy associated with proteasome inhibitors. Clin. Kidney J..

[24] Hobeika L., Self S.E., Velez J.C. (2014). Renal thrombotic microangiopathy and podocytopathy associated with the use of carfilzomib in a patient with multiple myeloma. BMC Nephrol..

[25] Chen Y., Ooi M., Lim S.F., Lin A., Lee J., Nagarajan C., Phipps C., Lee Y.S., Grigoropoulos N.F., Lao Z. (2016). Thrombotic microangiopathy during carfilzomib use: Case series in Singapore. Blood Cancer J..

[26] Lendvai N., Hilden P., Devlin S., Landau H., Hassoun H., Lesokhin A.M., Tsakos I., Redling K., Koehne G., Chung D.J. (2014). A phase 2 single-center study of carfilzomib 56 mg/m2 with or without low-dose dexamethasone in relapsed multiple myeloma. Blood J. Am. Soc. Hematol..

[27] Terao T., Tsushima T., Miura D., Ikeda D., Fukumoto A., Kuzume A., Tabata R., Narita K., Takeuchi M., Matsue K. (2022). Carfilzomib-induced thrombotic microangiopathy is underestimated in clinical practice: A report of five patients and literature review. Leuk. Lymphoma.

[28] Rassner M., Baur R., Wäsch R., Schiffer M., Schneider J., Mackensen A., Engelhardt M. (2021). Two cases of carfilzomib-induced thrombotic microangiopathy successfully treated with Eculizumab in multiple myeloma. BMC Nephrol..

[29] Darwin A., Malpica L., Dhanoa J., Hashmi H. (2021). Carfilzomib-induced atypical haemolytic uraemic syndrome: A diagnostic challenge and therapeutic success. BMJ Case Rep..

[30] Gavriilaki E., Peffault de Latour R., Risitano A.M. (2021). Advancing therapeutic complement inhibition in hematologic diseases: PNH and beyond. Blood.

[31] Blasco M., Martínez-Roca A., Rodríguez-Lobato L.G., Garcia-Herrera A., Rosiñol L., Castro P., Fernández S., Quintana L.F., Cibeira M.T., Bladé J. (2021). Complement as the enabler of carfilzomib-induced thrombotic microangiopathy. Br. J. Haematol..

[32] Gavriilaki E., Chrysanthopoulou A., Sakellari I., Batsis I., Mallouri D., Touloumenidou T., Papalexandri A., Mitsios A., Arampatzioglou A., Ritis K. (2019). Linking Complement Activation, Coagulation, and Neutrophils in Transplant-Associated Thrombotic Microangiopathy. Thromb. Haemost..

[33] Gavriilaki E., Anagnostopoulos A., Mastellos D.C. (2019). Complement in Thrombotic Microangiopathies: Unraveling Ariadne’s Thread into the Labyrinth of Complement Therapeutics. Front. Immunol..

[34] Portuguese A.J., Lipe B. (2018). Carfilzomib-induced aHUS responds to early eculizumab and may be associated with heterozygous CFHR3-CFHR1 deletion. Blood Adv..

[35] Hageman G.S., Hancox L.S., Taiber A.J., Gehrs K.M., Anderson D.H., Johnson L.V., Radeke M.J., Kavanagh D., Richards A., Atkinson J. (2006). Extended haplotypes in the complement factor H (CFH) and CFH-related (CFHR) family of genes protect against age-related macular degeneration: Characterization, ethnic distribution and evolutionary implications. Annu. Med..

[36] Gavriilaki E., Asteris P.G., Touloumenidou T., Koravou E.E., Koutra M., Papayanni P.G., Karali V., Papalexandri A., Varelas C., Chatzopoulou F. (2021). Genetic justification of severe COVID-19 using a rigorous algorithm. Clin. Immunol..

[37] Asteris P.G., Gavriilaki E., Touloumenidou T., Koravou E.E., Koutra M., Papayanni P.G., Pouleres A., Karali V., Lemonis M.E., Mamou A. (2022). Genetic prediction of ICU hospitalization and mortality in COVID-19 patients using artificial neural networks. J. Cell. Mol. Med..

[38] Moscvin M., Liacos C.I., Chen T., Theodorakakou F., Fotiou D., Regan E., Kastritis E., Dimopoulos M.A., Richardson P.G., Bianchi G. (2021). Mutations in the Alternative Complement Pathway in Multiple Myeloma Patients with Carfilzomib-Induced Thrombotic Microangiopathy. Blood.

[39] Feng S., Eyler S.J., Zhang Y., Maga T., Nester C.M., Kroll M.H., Smith R.J., Afshar-Kharghan V. (2013). Partial ADAMTS13 deficiency in atypical hemolytic uremic syndrome. Blood J. Am. Soc. Hematol..

[40] Chaturvedi S., Braunstein E.M., Yuan X., Yu J., Alexander A., Chen H., Gavriilaki E., Alluri R., Streiff M.B., Petri M. (2020). Complement activity and complement regulatory gene mutations are associated with thrombosis in APS and CAPS. Blood J. Am. Soc. Hematol..

